# Diagnostic Accuracy of Salivary Thiols Point‐of‐Care Test for Detecting Subgingival Dysbiosis in Adults: A Cross‐Sectional Study

**DOI:** 10.1002/cre2.70485

**Published:** 2026-10-15

**Authors:** Ahmed Khocht, Thomas E. Rams, Tsute Chen, Jon B. Suzuki

**Affiliations:** ^1^ Loma Linda University Loma Linda California USA; ^2^ Temple University Philadelphia Pennsylvania USA; ^3^ The ADA Forsyth Institute Somverville Massachusetts USA

**Keywords:** biofilms, dental plaque, microbial ecology, periodontal diseases, periodontitis, point‐of‐care test, saliva, sulfide

## Abstract

**Objectives:**

Many bacteria implicated in the pathogenesis of periodontitis produce thiol compounds containing sulfhydryl (‐SH) groups. This cross‐sectional study used a prototype chromogenic test strip to investigate the relationship between whole saliva ‐SH levels and subgingival bacterial species in subjects with and without periodontal disease.

**Materials and Methods:**

Ninety‐six subjects were enrolled. Full‐mouth comprehensive clinical periodontal evaluations were performed. Subjects were divided into 3 groups: periodontally healthy, gingivitis, and periodontitis. Subgingival plaque samples were collected and analyzed for their content of 40 bacterial species using checkerboard DNA–DNA hybridization. Subgingival dysbiosis severity was determined with a modified Subgingival Microbial Dysbiosis Index (mSMDI). Salivary thiol levels were determined semi‐quantitatively with a chromogenic strip dipped in pooled saliva on the tongue surface.

**Results:**

Low and high strip scores were uniquely associated with contrasting groups of subgingival bacteria. Individuals with high strip scores harbored increased levels of *P. gingivalis, P. intermedia, T. forsythia, P. nigrescens, E. nodatum, and T. denticola* as well as higher mSMDI scores than individuals with low strip scores. Furthermore, strip scores showed high specificity (93%) in correctly identifying individuals with normobiotic subgingival microbiota.

**Conclusions:**

Low salivary thiol levels are useful indicators of subgingival normobiosis.

## Clinical Relevance

**Scientific Rationale for Study:** Salivary point‐of‐care test may potentially enable rapid and non‐invasive detection of subgingival dysbiosis, and thiols are emerging as a promising diagnostic biomarker. However, the efficacy of salivary thiol levels in detecting subgingival microbial dysbiosis has not been previously evaluated.

**Principal Findings:** Salivary thiol levels showed a relevant positive relation with several subgingival microbial species in the so‐called orange and red complexes and utility for ruling out subgingival dysbiosis tendency.

**Practical Implications:** Salivary chromogenic strip assessments provide a simple, non‐invasive, and user‐friendly option for ruling out subgingival dysbiosis and may be useful in combination with other diagnostic assessments for monitoring periodontal health.

## 1 Introduction

The role of subgingival bacterial dysbiosis in the initiation and progression of periodontal diseases is well documented (Hajishengallis et al. 2012, 2015, 2020). Dysbiotic bacterial alterations that involve increased levels of *Porphyromonas gingivalis, Treponema denticola,* and *Tannerella forsythia* in subgingival plaque have been associated with increased risk for destructive periodontitis (Socransky et al. 1998; Darveau 2010; Suzuki et al. 2013). These three bacterial species, collectively known as the red complex, have been proposed as a pathogenic consortium promoting further dysbiosis of the subgingival microbial community (Holt et al. 2000).

A conventional periodontal evaluation does not allow for the assessment of the microbial profile involved with destructive periodontal changes. Having knowledge of the subgingival microbial composition would enhance diagnosis and management of periodontal disease. Although a few chairside point‐of‐care tests are available to evaluate the subgingival microbiome, these tests are limited to a small number of bacterial species, and some require specialized equipment (Wetzel et al. 1991; Hemmings et al. 1997; Andrade et al. 2010; Arweiler et al. 2020).

Many of the bacteria involved with periodontal disease are known to produce thiol compounds as end products of metabolism (Persson 1992; Langendijk‐Genevaux et al. 2001; Torresyap et al. 2003). Thiol‐producing microbial species degrade proteins with sulfate‐containing amino acids as an energy source. The main thiol‐containing end products produced by oral bacteria include hydrogen sulfide, methyl mercaptan, dimethyl sulfide, and dimethyl disulfide (Hampelska et al. 2020). A number of in vitro experiments have indicated that thiol compounds may play an important role in the pathogenesis of periodontitis, by impairing epithelial and fibroblast cell function (DeCarlo et al. 1998), enhancing oxidative stress toxicity (Yagi et al. 2021), up‐regulating IL‐8 expression in gingival and oral epithelial cells (Chen et al. 2010), facilitating the penetration of bacterial agents (such as lipopolysaccharides) into gingival tissues (Ratcliff and Johnson 1999), and enhancing interleukin‐1 production (Deschner et al. 2003), subsequently enhancing the production of matrix metalloproteinases and prostaglandin E‐2, which are involved in soft and hard periodontal tissue damage (Ratcliff and Johnson 1999).

Thiol assessment is difficult and usually involves assessing volatile sulfur compounds with complicated and/or expensive equipment (Persson 1992; Langendijk‐Genevaux et al. 2001; Torresyap et al. 2003). Previously, we described a prototype dry biochemical thiol detection test which employs a strip with a porous pad impregnated with a chromogenic reagent that detects thiol compounds dissolved in oral fluids (Khocht et al. 2013). The strip provides a semi‐quantitative visual assessment of thiol compounds (Ellman 1958). We showed that periodontitis patients have elevated thiol compound levels in whole saliva and that salivary thiol compound levels are positively associated with clinical measures of periodontitis (Khocht et al. 2013).

In this study, we hypothesized that salivary thiol levels are related to dysbiotic changes in the subgingival microbial composition and would align with increased levels of red complex and other periodontopathic bacteria. The objectives of this study were to investigate the relationship between whole saliva thiol levels determined by the chromogenic thiol detection strip with a modified version of the Subgingival Microbiome Dysbiosis Index (mSMDI) and Socransky's subgingival bacterial complexes, including the red complex in individuals with and without periodontal disease.

## 2 Materials and Methods

This study was conducted at Temple University School of Dentistry, Philadelphia, Pennsylvania, 2010–2012. Subject recruitment/enrollment and inclusion/exclusion criteria were previously described (Khocht et al. 2013). A total of 100 subjects were screened, 4 were disqualified for not meeting all study criteria, and 96 were enrolled (Figure 1). Informed consent was obtained from all subjects.

**Figure 1:**
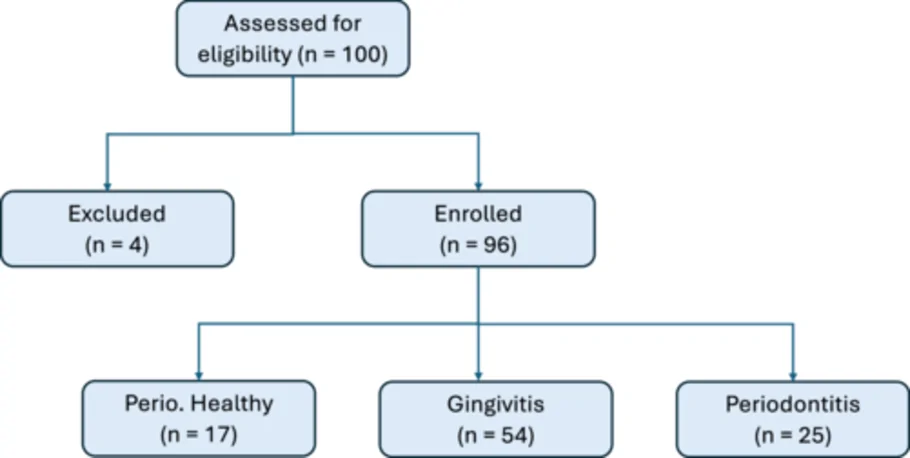
Participant flow diagram.

### 2.1 Subject Qualification

Inclusion criteria for this study required patients to be in good general health, 21 years of age or older, able to communicate in English, and to have a minimum of 12 natural usable teeth. Any of the following conditions excluded subjects from eligibility for study participation: medical conditions with a known impact on periodontal status (such as diabetes), medical conditions requiring prophylactic antibiotic coverage, medical conditions that impact salivary function, the use of anti‐inflammatory medications, current participation in a dental plaque/gingivitis clinical trial, pregnancy, gross dental caries or other significant oral diseases, and wearing a fixed orthodontic appliance.

### 2.2 Cigarette Smoking Questionnaire

The following information was collected: current or past smoker, number of cigarettes smoked per day, and for how many years.

### 2.3 Oral/Periodontal Assessments

All subjects received a comprehensive oral/periodontal evaluation including probing measurements. A single experienced dental examiner (A.K.) performed all dental exams. The examiner was calibrated and standardized in the use of the clinical evaluation measures employed in the study. The intra‐examiner probing measures agreement (probing differences within 1 mm) was 98%.

The examiner recorded the plaque index (PI) and the gingival index (GI) around all present teeth (Löe 1967). A conventional periodontal probe (Michigan‐O Probe with Williams markings, Hu‐Friedy, Chicago, IL) was used for all probing measurements. Probing depth (PD) was recorded on six sites per tooth. The position of the gingival margin (GM) to the cemento‐enamel junction was recorded at the same six sites per tooth. Periodontal attachment level (AL) was calculated per the formula AL = PD + GM.

Based on periodontal evaluations, subjects were assigned to one of the following periodontal diagnosis groups (Caton et al. 2018): periodontally healthy subjects (no loss of clinical attachment, PD < 4 mm and BOP < 10%), gingivitis subjects (no loss of clinical attachment, PD < 4 mm and BOP > 10%) and periodontitis subjects (for maximum contrast, stage III, CAL > 4 mm with increased pocket depth and associated BOP).

### 2.4 Thiol Detecting Strip

The thiol detecting strip directly measures the concentration of thiols in saliva and provides a visual signal related to the thiol concentration. The pad on the strip contains a thiol detection reagent that, in the presence of thiols, produces the yellow color of thionitrobenzoate (Ellman 1958). The intensity of the color developed on the strip is directly related to the concentration of thiols in the sample. The strip is formulated to detect thiol concentrations ranging from 0 to 600 μM (Khocht et al. 2013). A comparator color chart with color scores ranging from 0 to 10 was used to score the color change. A score of 0 indicates no color change, and a score of 10 indicates thiol concentration = > 600 μM. The comparator color chart allows a rapid visual scoring of the thiol content in the sample without need for additional equipment. Safety studies, stability of reagent, sensitivity, selectivity, and reliability of the quantitative color responses were previously reported (Burgess‐Cassler and Haley 2009).

### 2.5 Saliva Sampling

Prior to initiating the periodontal measures, subjects were instructed to close their mouth, rub their tongue against the lingual aspects of the gingival margins, pool their saliva on the tongue surface, and open their mouth. The padded end of the strip was dipped in the pooled saliva on the tongue surface without rubbing against the tongue (Khocht et al. 2013).

### 2.6 Strip Scoring

After saliva collection, each strip was allowed 30 s for the color reaction to develop (Khocht et al. 2013). A team of two blinded (unaware of clinical periodontal status) evaluators performed strip scoring. Strip score was determined by comparing the intensity of color change on the strip to the color chart (Khocht et al. 2013). While holding the strip near the colors available on the comparison color chart, the score of the color closest to the color on the strip was selected as the strip score. If the color change on the strip was not uniform, scoring was based on the most intense color seen on the pad. The two evaluators independently scored each strip. The average of the two scores was used for data analysis.

To minimize intra‐evaluator and inter‐evaluator variability, the two strip evaluators were calibrated to recognize and agree on the intensity of the color change on the strip and the corresponding color score. Calibration data showed intra‐evaluator agreement (same score between first and second readings) for evaluator A was 98% and evaluator B was 97%. Inter‐evaluator agreement (same score between first and second evaluator) was 94%.

### 2.7 Microbiological Evaluations

Three sites per subject were sampled for microbial analysis after completion of the clinical evaluations. In periodontitis subjects, three interproximal sites with PD > 5 mm were randomly chosen from three different teeth in separate quadrants. In non‐periodontitis subjects, three interproximal sites with PD < 4 mm were randomly chosen from three different teeth in separate quadrants. After removal of supragingival plaque and isolation to prevent saliva contamination, subgingival plaque samples were collected with a sterile Gracey curette from the selected sites. All subgingival samples per subject were pooled into a single Eppendorf tube containing 450 µL of TE buffer (10 mM Tris‐HCl, 1 mM EDTA, pH 7.6), followed by the addition of 300 µL of 0.5 M NaOH. The samples were immediately frozen at −70°C till microbiological analysis.

The presence and levels of 40 bacterial species were determined in each pooled subgingival plaque sample using species‐specific whole genomic DNA probes and a modification (Haffajee et al. 1997) of the checkerboard DNA–DNA hybridization technique (Socransky et al. 1994). Laboratory analyses were conducted for all samples together immediately after the conclusion of the study.

### 2.8 Calculation of Dysbiotic Tendencies in Subgingival Plaque

As a measure of subgingival dysbiosis, we used a modified version of the SMDI (Yakar et al. 2025). The following discriminating species were categorized as either normobiotic (*Actinomyces naeslundii, Veillonella parvula, Streptococcus sanguinis*) or dysbiotic (*Streptococcus constellatus, Porphyromonas gingivalis, Eubacterium nodatum, Fusobacterium nucleatum ssp. vincentii, Tannerella forsythia, Treponema denticola, and Treponema socranskii*). We applied the centered log ratio (clr) transformation to the percent DNA of these species, using the formula clr= log(*x*/*G*), where *x* represented the percent DNA of the individual bacterial species, and *G* was the geometric mean of the total percent DNA of all 40 species. After clr transformation, mSMDI was calculated as the mean clr of dysbiotic species minus the mean clr of normobiotic species.

### 2.9 Data Analysis

A priori power analysis, with a significance level of *α* = 0.05 and a desired power of 0.80, indicated that a sample size of approximately 17 participants per group would be needed to detect a statistically significant difference in strip scores between groups in a one‐way analysis of variance (ANOVA) test (Khocht et al. 2013).

The average for each clinical measure was computed per subject and then averaged across subjects in each group. The percentage of DNA probe counts comprised by each of the tested bacterial species in pooled subgingival samples was determined for each study subject, with species proportions then averaged across subjects in each of the three study subject groups. To assess for statistically significant between‐group differences in demographic, clinical and strip score parameters, ANOVA was used on mean values, and chi‐square tests on proportional data. Bacterial measures were skewed. Significant differences in mSMDI scores and bacterial species proportions among study subject groups were evaluated using the Kruskal–Wallis test. Microbial comparisons were adjusted for multiple testing using the False Discovery Rate method (Benjamini and Hochberg 1995). The zero‐order associations between numerical variables were quantified with Spearman coefficients (*ρ*).

For analysis purposes, strip scores, mSMDI scores and red group complex totals were dichotomized. Strip scores ≥ 3, mSMDI scores > 0, and red group species > 75th percentile (> 10% of total DNA) were deemed elevated. Nominal logistic regression analysis (unadjusted and adjusted for cigarette smoking) was used to calculate the odds ratio between elevated red complex bacterial species and elevated mSMDI scores with elevated strip scores. Sensitivity (true positive rate) and specificity (true negative rate) were assessed for strip score ability to identify individuals with subgingival bacterial dysbiosis (mSMDI scores > 0) and for harboring high levels (exceeding 75th percentile) of red complex bacterial species in the subgingival plaque.

A type 1 error rate of 5% was used to assess statistical significance.

## 3 Results

Demographic, cigarette smoking, and clinical data were previously presented (Khocht et al. 2013). Table 1a summarizes demographic and cigarette smoking data of subjects by periodontal status. Periodontitis subjects were older than both the periodontally healthy and gingivitis subjects. Gender distribution was similar between the three groups. Periodontitis subjects had a lower percentage of Caucasians and a higher percentage of smokers than the non‐periodontitis subjects. Table 1b summarizes the clinical measures by periodontal status. ANOVA showed significant differences between the three groups for percent of missing teeth and all periodontal parameters, including attachment level, pocket depth, plaque index, gingival index, and bleeding on probing. The periodontitis subjects were higher on all clinical parameters than gingivitis and healthy subjects.

**Table 1:** Demographic and clinical data.

	Healthy	Gingivitis	Periodontitis	*p*‐value
	*N* = 17	*N* = 54	*N* = 25	
**a. Demographic data by subject group**				
Age	32.17 (3.0)	30.61 (1.68)	52.16 (2.47)	0.0001
% Males	70	50	44	0.21
% White	64	68	28	0.002
% Smokers	35	18	64	0.0004
**b. Clinical periodontal data by subject group**				
PI	0.31 (0.09)	0.55 (0.05)	1.11 (0.07)	0.0001
GI	0.31 (0.04)	0.48 (0.02)	0.78 (0.04)	0.0001
% BOP	4.56 (4.32)	13.08 (2.42)	38.15 (3.56)	0.0001
PD mm	2.33 (0.12)	2.40 (0.06)	3.38 (0.09)	0.0001
CAL mm	0.17 (0.29)	0.10 (0.06)	3.06 (0.24)	0.0001
% sites ≥ 4 mm	0	0	21.68	
Missing teeth%	3.37 (1.49)	6.72 (2.66)	18.85 (2.19)	0.0001

*Note:* Data presented as mean (SE) or percentage. *p*‐value gives the probability that the groups differ in either an ANOVA test or chi‐square test, for means or proportions, respectively.

### 3.1 Chromogenic Strip Scores

The color score range of the chromogenic strip test was 1 to 6. The majority of subjects (*n* = 84) had low strip scores (range: 1–2) and only 12 subjects had strip scores 3 or higher (range: 3–6). The proportion of strip scores significantly differed by subject group (Figure 2). The periodontally healthy subject scores were all in the low 1–2 range, approximately 4% of the gingivitis subjects showed a score of 3, and approximately 40% of the periodontitis subject showed scores in the 3–6 range.

**Figure 2:**
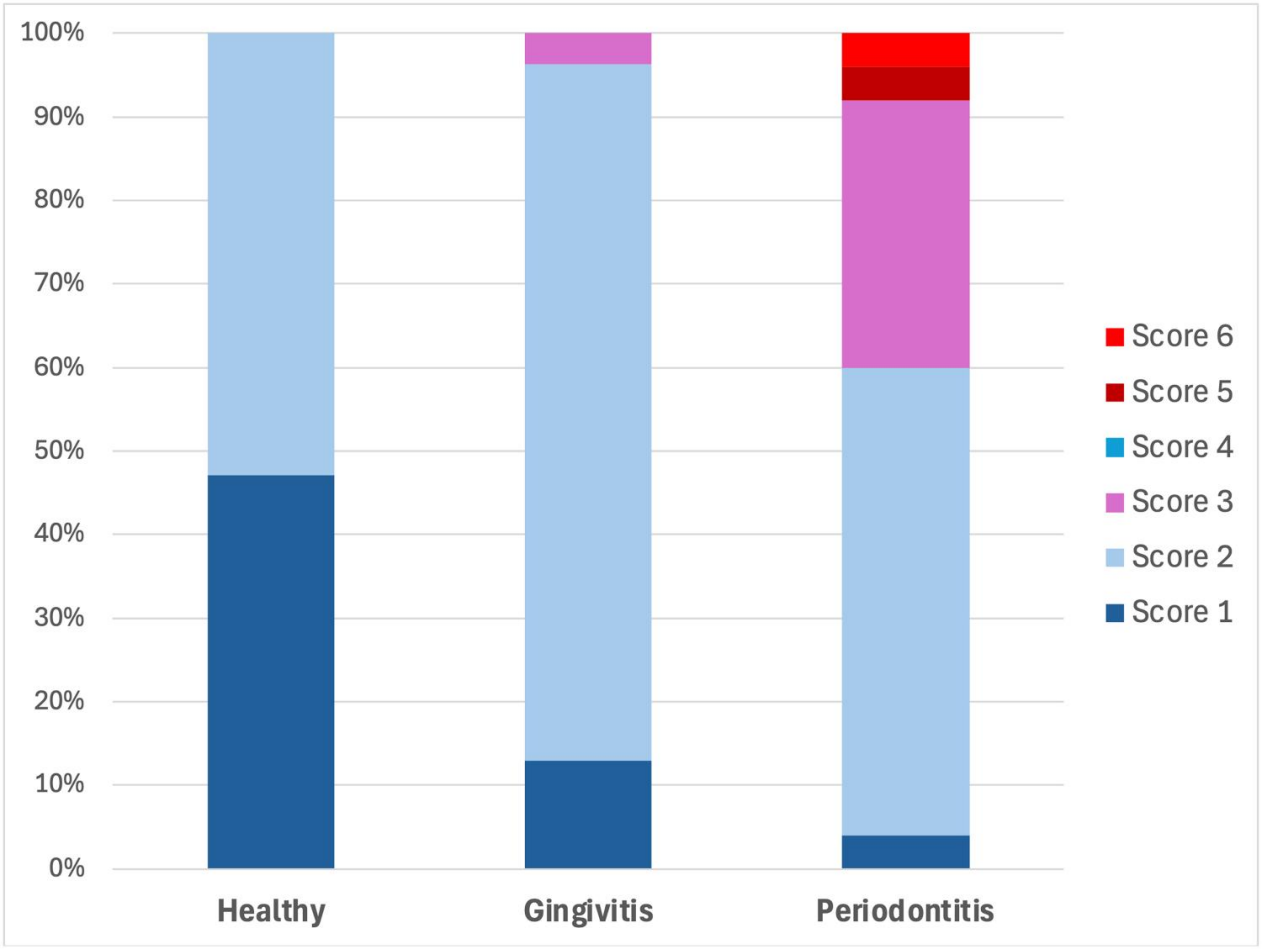
Proportion of strip scores by periodontal status. Data are presented as percentage. Chi‐square test for proportions showed significant differences in strip scores among the 3 groups (Likelihood ratio 32.24, *p* < 0.0001).

### 3.2 Microbiological Findings

#### 3.2.1 Subgingival Bacterial Levels by Subject Group

As would be expected, the mean ×10^5^ (SE) total DNA probe counts were significantly higher in the periodontitis subjects 98.82 (11.03) than in the healthy 44.95 (13.37) and gingivitis 48.61 (7.50) subjects, *p* = 0.002. Figure 3 presents the mean percentage of the DNA probe counts of the 40 test species by subject group. The species were ordered according to the microbial complexes described by Socransky et al. (1998). Five of the test species were found at significantly higher levels in the periodontitis subjects compared with the healthy and gingivitis subjects. These included members of the genera *Campylobacter rectus, Eubacterium nodatum, Prevotella nigrescens, T. forsythia*, and *P. gingivalis. A. naeslundii 2* levels were significantly elevated in periodontally healthy subjects, and *S. mitis* levels were significantly elevated in gingivitis subjects.

**Figure 3:**
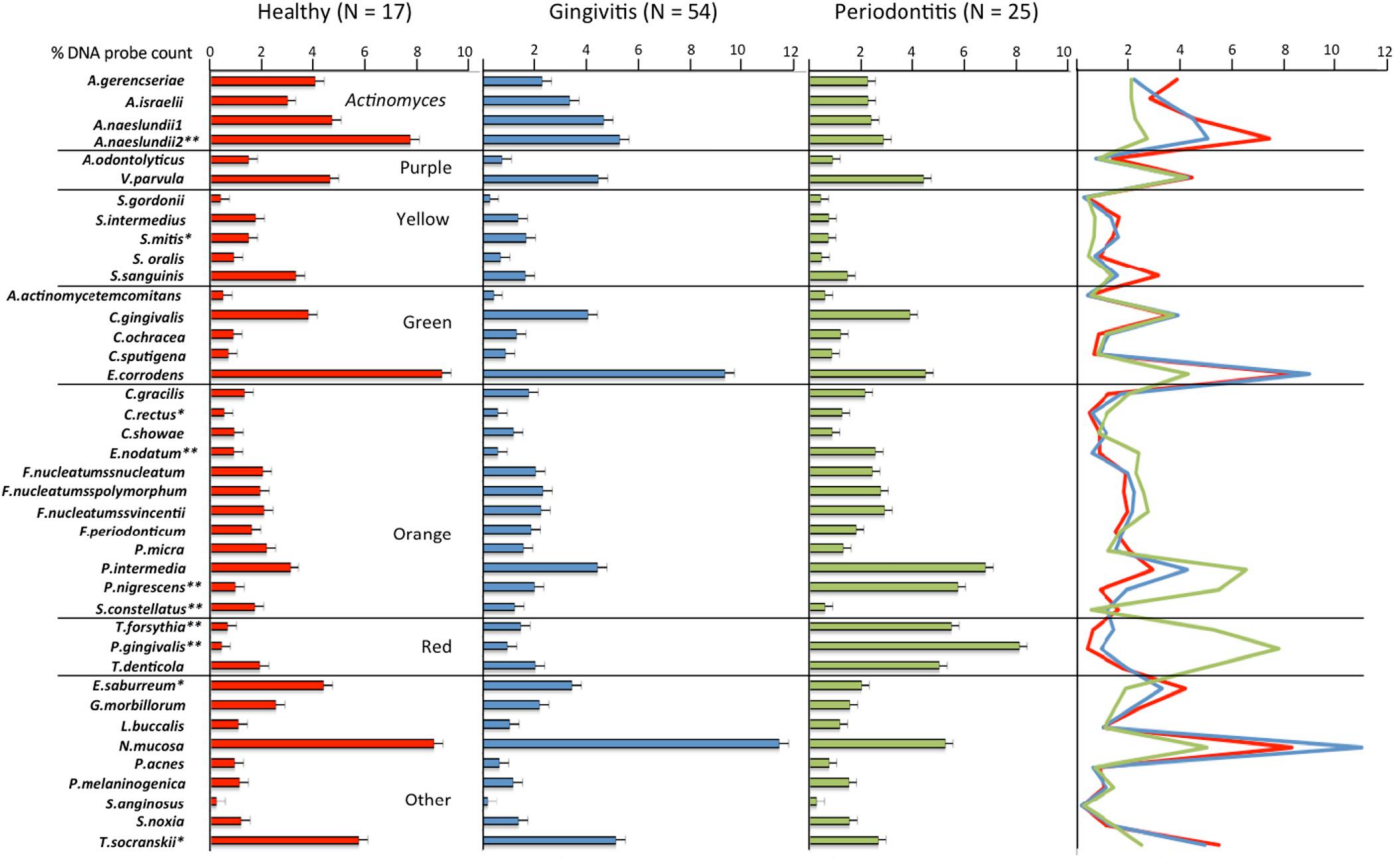
Bar chart of mean % of the DNA probe count of individual species in subgingival plaque samples taken from periodontally healthy, gingivitis, and periodontitis subjects. The bars represent the mean percentages and the whiskers the standard error of the mean. The % of the DNA probe count for each species was computed per subject and then averaged across subjects in each group. Significance of differences between groups was sought using the Kruskal–Wallis test, adjusted for 40 comparisons, *p < 0.05, **p < 0.01.

### 3.3 Validation of mSMDI Scores With Clinical Measures, Cigarette Smoking, Strip Scores and Red Complex Bacteria

For all subjects combined, mSMDI scores ranged from −3.2 (minimum) to 2.43 (maximum). Figure 4 presents mSMDI scores by subject group. Participants diagnosed with periodontitis showed significantly elevated mSMDI scores compared to those without periodontitis. Smokers exhibited significantly higher mSMDI scores than non‐smokers. Additionally, individuals with a high strip score (range: 3–6) exhibited significantly higher mSMDI scores than individuals with a low strip score (range: 1–2). Furthermore, Spearman correlation analysis indicated a positive association between mSMDI scores with gingival index (*ρ* = 0.33, *p* < 0.01), percentage of bleeding pockets (*ρ* = 0.44, *p* < 0.01), probing depths (*ρ* = 0.29, *p* < 0.01), clinical attachment levels (*ρ* = 0.44, *p* < 0.01), strip scores (*ρ* = 0.24, *p* < 0.05), and red complex bacteria totals (*ρ* = 0.81, *p* < 0.0001).

**Figure 4:**
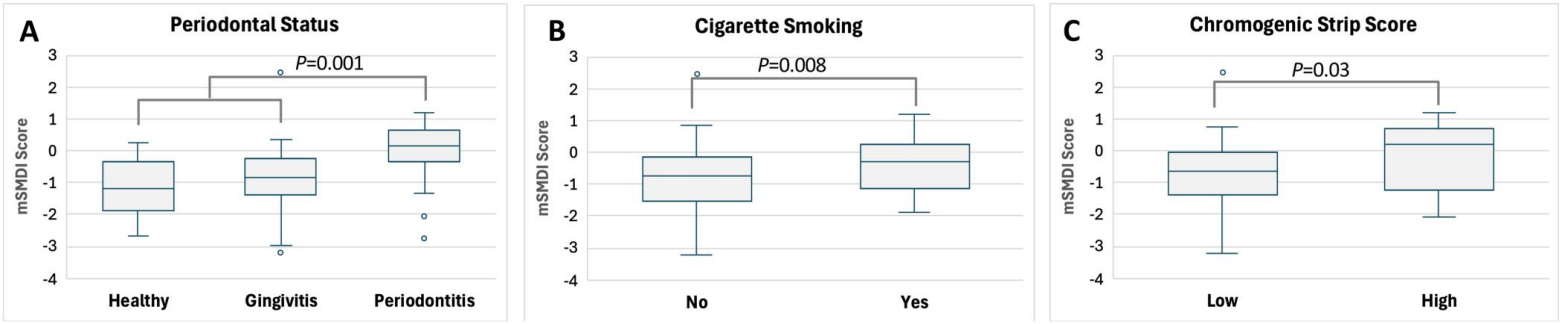
Box plots of the modified Subgingival Dysbiosis Index (mSMDI) by group categories. The bottom and top sides of each box represent the lower and upper quartiles, respectively. The line inside the box represents the median. The bottom and top whiskers represent the minimum and maximum values, respectively. Dots denote outliers. (A) Periodontal diagnosis: Kruskal–Wallis test showed statistically significant differences between the 3 periodontal groups (*χ* ^2^ ((2), *N* = 96) = 16.41, *p* = 0.0003). Post‐hoc comparisons using Dunn's method showed that the mSMDI was significantly higher in the periodontitis group than the health group (*p* = 0.001) and the gingivitis group (*p* = 0.001). (B) Cigarette smoking: Kruskal–Wallis test showed higher mSMDI scores in smokers than non‐smokers (*χ* ^2^ ((1), *N* = 96) = 6.90, *p* = 0.008). (C) Chromogenic strip scores: Kruskal–Wallis test showed higher mSMDI scores in individuals with high strip score than individuals with low strip score (*χ* ^2^ ((1), *N* = 96) = 4.76, *p* = 0.03).

### 3.4 Zero‐Order Associations Between Strip Scores With Individual Bacterial Species

The zero‐order correlations (Spearman *ρ*) between the mean percent of the DNA probe count of each of the microbial species with the strip scores are presented in Table 2. Several weak to moderate significant negative correlations were observed between the mean percent DNA counts of *Actinomyces gerencseriae, A. naeslundii, A. odontolyticus, Streptococcus intermedius, S. mitis, S. oralis, S. sanguinis, Fusobacterium periodonticum, Parvimonas micra, S. constellatus, Gemella morbillorum*, and *Cutibacterium acnes* (old name: *Propionibacterium acnes)* with the strip scores. Several weak to moderate significant positive correlations were observed between the mean percent DNA counts of *P. intermedia, P. nigrescens, T. forsythia, P. gingivalis* with the strip scores.

**Table 2:** Zero‐order correlations between strip scores and bacterial species % DNA count (all groups combined).

Complex	Species	Spearman *ρ*	*p*‐value
Actinomyces	***A. gerencseriae***	**−0.21**	**0.03**
	*A. israelii*	−0.11	0.26
	*A. naeslundii 1*	−0.13	0.19
	***A. naeslundii 2***	**−0.35**	**0.0005**
Purple	***A. odontolyticus***	**−0.35**	**0.0003**
	*V. parvula*	−0.06	0.57
Yellow	*S. gordonii*	−0.15	0.13
	***S. intermedius***	**−0.25**	**0.01**
	***S. mitis***	**−0.26**	**0.01**
	***S. oralis***	**−0.29**	**0.003**
	***S. sanguinis***	**−0.32**	**0.001**
Green	*A. actinomycetemcomitans*	0.00	0.96
	*C. gingivalis*	−0.07	0.48
	*C. ochracea*	0.05	0.66
	*C. sputigena*	0.07	0.47
	*E. corrodens*	−0.09	0.41
Orange	*C. gracilis*	0.11	0.28
	*C. rectus*	0.06	0.56
	*C. showae*	−0.15	0.16
	*E. nodatum*	0.11	0.29
	*F. nucleatumssnucleatum*	−0.15	0.14
	*F. nucleatumsspolymorphum*	−0.06	0.53
	*F. nucleatumssvincentii*	0.10	0.31
	***F. periodonticum***	**−0.21**	**0.04**
	***P. micra***	**−0.29**	**0.004**
	***P. intermedia***	**0.33**	**0.001**
	***P. nigrescens***	**0.33**	**0.001**
	***S. constellatus***	**−0.27**	**0.007**
Red	***T. forsythia***	**0.33**	**0.0009**
	***P. gingivalis***	**0.25**	**0.01**
	*T. denticola*	0.04	0.68
Other	*E. saburreum*	−0.19	0.06
	***G. morbillorum***	**−0.24**	**0.01**
	*L. buccalis*	0.01	0.92
	*N. mucosa*	−0.11	0.29
	***C. acnes***	**−0.26**	**0.01**
	*P. melaninogenica*	−0.04	0.72
	*S. anginosus*	0.04	0.70
	*S. noxia*	−0.06	0.59
	*T. socranskii*	−0.20	0.06

*Note:* Statistically significant associations are bolded.

### 3.5 Subgingival Microbial Profile by Strip Score Category (Low vs. High)

The differences in the mean percentage of the DNA probe counts of the microbial species between low strip score (range: 1–2) and high strip score (range: 3–6) subject categories were computed (Figure 5). Fourteen of the test species were found at higher levels in the high strip score subjects when compared to the low strip score subjects. The top six species that showed the highest differences were, in the following order, *P. gingivalis, P. intermedia, T. forsythia, P. nigrescens, E. nodatum,* and *T. denticola*. All these species belonged to the red and orange complexes.

**Figure 5:**
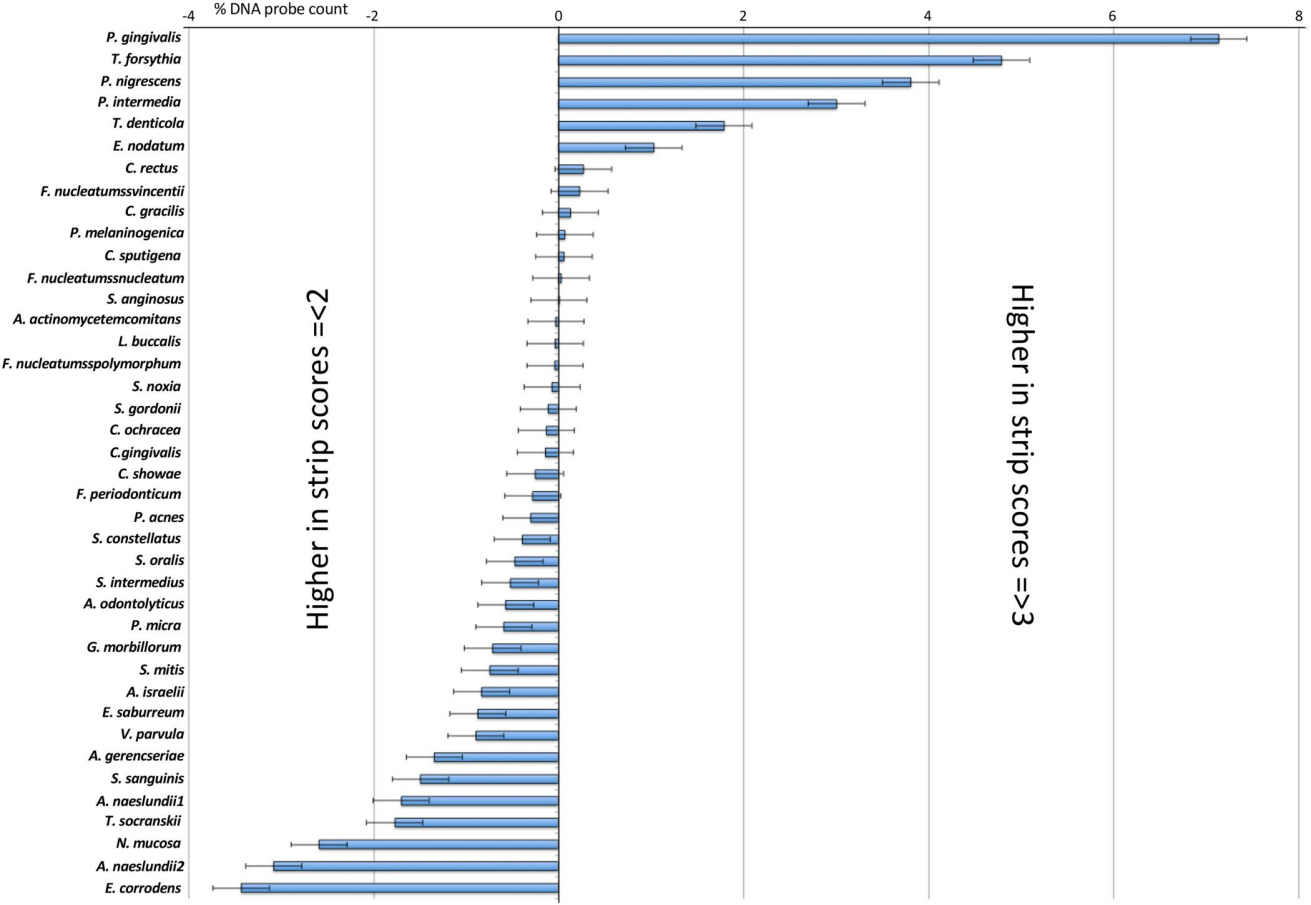
Bar chart of the mean differences in % DNA probe counts of individual species between low and high strip score subject categories. All groups combined. The bars represent the mean difference in probe counts, and the whiskers indicate the standard error of the mean. Mean differences in % DNA counts for each species were computed between low strip score subjects and high strip score subjects. The species were ordered according to the greatest difference between low strip score and high strip score categories. Bars to the right of 0 represent species that were higher in subjects with high strip scores.

### 3.6 Strength and Accuracy of Chromogenic Strip Scores in Relation to Bacterial Measures

Table 3a examines the strength of the association (odds ratio) between positive strip scores with subgingival red complex bacteria (grouped together) and mSMDI scores. Univariate regression analysis showed that individuals with elevated strip scores (range: 3–6) have significantly higher odds of increased levels of red complex bacteria and dysbiotic tendency than individuals with low strip scores (range: 1–2). Adjusting for cigarette smoking (potential confounder) confirmed the univariate association, meaning that individuals with elevated strip scores despite smoking are still more likely to have increased levels of red complex bacteria as well as dysbiosis tendencies. Table 3b examines the accuracy (sensitivity/specificity analyses) of strip scores in detecting individuals with elevated subgingival red complex bacteria and dysbiosis tendencies. Chromogenic strip score responses were identical for both red complex bacteria (grouped together) and for mSMDI scores. Overall, strip scores showed low sensitivity and high specificity in correctly identifying individuals with elevated subgingival red complex bacteria and dysbiosis tendencies.

**Table 3:** Strength and accuracy of chromogenic strip scores in detecting bacterial measures (elevated red complex bacteria and elevated mSMDI scores), all groups combined.

	Unadjusted			Adjusted		
	OR	95% CI	*p*‐value	OR	95% CI	*p*‐value
**a. Odds ratio**						
Red complex	5.94	1.67, 21.19	0.005	5.37	1.45, 19.78	0.01
mSMDI scores	5.95	1.69, 22.51	0.005	5.37	1.44, 21.45	0.01

		Red complex			mSMDI score	
		Positive > 75th %ile	Negative ≤ 75th %ile		Positive > 0	Negative ≤ 0
**b. Sensitivity/Specificity**						
Strip score	Positive ≥ 3 score	7	5	Positive ≥ 3 score	7	5
	Negative < 3 score	16	68	Negative < 3 score	16	68
		Sensitivity 30%	Specificity 93%		Sensitivity 30%	Specificity 93%

*Note:* (a) Odds ratio for positive strip scores (≥ 3) in relation to elevated total red complex species (cutoff level > 75th percentile) and elevated mSMDI (cutoff score > 0). Data are presented unadjusted and adjusted for history of cigarette smoking. Nominal logistic regression analysis showed that positive strip scores (≥ 3) were associated with higher odds of elevated red complex species (OR = 5.94) and higher odds of dysbiotic tendency (OR = 5.95). Adjusting for cigarette smoking confirmed the univariate analysis. (b). Sensitivity/specificity of strip scores on detection of elevated red complex bacteria and bacterial dysbiosis tendencies. Data are presented as number of subjects. For both red complex bacteria and mSMDI, chromogenic strip score distributions were similar and showed low sensitivity (30%) and high specificity (93%) in correctly identifying individuals with either elevated levels of red complex bacteria or dysbiotic tendencies. Likelihood ratio of chromogenic test results: *LR*+ = 4.41 (small); *LR*− = 0.74 (large), suggesting strong utility for ruling out elevated levels of red complex bacteria and dysbiosis tendency.

## 4 Discussion

Our study is the first to assess the relationship between salivary thiols with a quantifiable measure of subgingival microbial dysbiosis using a chromogenic thiol strip. The results showed a relevant positive relation between salivary thiol scores with several subgingival microbial species in the so‐called orange and red complexes and with dysbiotic subgingival plaque tendencies. Low and high strip scores were uniquely associated with contrasting groups of subgingival bacteria. The findings also showed a trade‐off between the test's specificity and sensitivity. The test's ability to correctly identify individuals harboring low levels of red complex bacteria or normobiotic mSMDI scores (specificity) inversely associated with its ability to correctly identify individuals harboring high levels of red complex bacteria or dysbiotic mSMDI scores (sensitivity).

For the purposes of this study, we employed checkerboard DNA–DNA hybridization for bacterial assessments. Although more thorough bacterial assessment techniques are available, such as 16S rRNA gene sequencing and other next‐generation sequencing approaches, we were mainly interested in a limited group of sulfide‐producing bacterial species previously identified with the checkerboard DNA–DNA hybridization technique (Torresyap et al. 2003).

The microbial species that showed increased levels in individuals with elevated salivary thiol scores included all three red complex species (*P. gingivalis, T. forsythia, T. denticola*) and three species from the orange complex (*P. intermedia, P. nigrescens, E. nodatum*). All these species are known to be high thiol producers (Torresyap et al. 2003). Furthermore, *T. forsythia* and *P. gingivalis* are designated periodontal pathogens (Proceedings of the 1996 World Workshop in Periodontics 1996). Sulfur‐producing bacteria differ in the levels and nature of the thiol‐containing end products they produce (Persson et al. 1990; Nakano et al. 2002; Yoshida et al. 2009; Hampelska et al. 2020). Thus, some thiol‐containing end products, because of their low levels or different nature, may not be detected by the chromogenic strip. This may explain why not all sulfur‐producing bacteria assessed in this study were associated with the strip scores.

The salivary sampling used in this study does not allow for the determination of the sources of thiols detected by the strip. The positive association noted between five thiol‐producing subgingival microbial species and the strip scores suggests that microbial thiol end‐products produced subgingivally exit the subgingival environment and dissolve in salivary fluid. It is also likely that other microbial communities in the oral cavity, like on the dorsum of the tongue and other mucosal surfaces, may also harbor thiol‐producing bacteria that have contributed to salivary thiol levels. Indeed, there is a positive relation between subgingival microbial species and tongue microbial species (Tanner et al. 2006); the associations noted between strip scores and subgingival microbial species might be indirectly mediated by thiol‐producing bacteria on the dorsum of the tongue. Another source of thiols is cigarette smoking (Christen 1992; Rosenberg 1996). In this study, the effect of cigarette smoking on strip scores was negligible. This suggests that thiols contained in cigarette smoke are short‐lived in the oral environment.

Overall, the strip score observations mirrored the subgingival microbial profile of the three periodontal subject groups in this study. The lower counts of the orange and red microbial species in both the periodontally healthy subjects and the gingivitis subjects may explain their comparable low thiol strip scores; and the higher counts of the orange and red microbial species in the periodontitis group may explain why their thiol strip scores were significantly higher than the other two groups. However, strip scores failed to identify some individuals with highly elevated subgingival red complex species. This may suggest that not all subgingival bacterial communities with elevated red complex species are actively producing thiol products. Alternatively, salivary thiol levels may not accurately align with subgingival microbiota and may explain the low sensitivity of the chromogenic strip.

Our point‐of‐care test confirmed and expanded upon previous research utilizing more sophisticated equipment to investigate the relation between volatile sulfur compounds and periodontopathic bacteria in the periodontal pocket. Previous studies utilizing a sulfide sensor probe showed a positive correlation between subgingival sulfides with BANA scores representing the three red complex bacteria (Morita and Wang 2001) as well as sulfide‐producing species assessed with checkerboard DNA‐DNA hybridization (Torresyap et al. 2003).

Although this study has provided insights into the relationship between salivary thiols and subgingival microbiota, the limited test sensitivity reduces its utility as a screening or diagnostic tool. Due to strip design, direct assessment of subgingival thiol measures was not feasible. The sources of thiols in saliva and salivary microbial assessments were not investigated. Furthermore, subgingival microbial sampling was limited to only three sites, and microbial assessments were limited to only 40 species. The three subject groups differ substantially in smoking prevalence, age, and racial composition, which may have influenced their microbial composition. Future research should focus on ways to improve the test's specificity. Strip design modification may enable direct assessment of subgingival thiol levels in relation to subgingival bacteria and improve the test's effectiveness. Longitudinal studies to evaluate the utility of salivary thiol assessments in managing periodontitis patients may help improve periodontal care of periodontitis patients after completion of initial periodontal therapy.

In conclusion, although the point‐of‐care salivary thiol assessments showed high specificity and were significantly associated with the subgingival levels of several major periodontopathic bacterial species, they did not fully reflect the levels of the entire subgingival microbial community. Nevertheless, the findings suggest that low salivary thiol levels are reliable indicators of normobiotic subgingival flora and low levels of periodontal pathogens.

## Author Contributions

**Ahmed Khocht:** study conception and design, acquisition of data, statistical analysis and interpretation of results, manuscript preparation. **Thomas E. Rams:** study development and administration, critically revised manuscript. **Tsute Chen:** microbial analysis, interpretation of microbial data, critically revised manuscript. **Jon B. Suzuki:** study development and administration, critically revised manuscript. All authors reviewed and approved the final version of the manuscript.

## Ethics Statement

The study was approved by Temple University Institutional Review Board (IRB #11275: TOPAS: Test Values in Human Periodontal Health, Gingivitis, and Chronic Periodontitis). This research was conducted in accordance with the principles embodied in the Declaration of Helsinki and in accordance with local statutory requirements. All participants gave written informed consent to participate in the study.

## Conflicts of Interest

The authors declare no conflicts of interest.

## Data Availability

The data published in this study are available upon reasonable request from author.
